# Viral contamination in cell culture: analyzing the impact of Epstein Barr virus and Ovine Herpesvirus 2

**DOI:** 10.3389/fmicb.2025.1442321

**Published:** 2025-02-25

**Authors:** Iman M. Bastawecy, Mohamed Abdelmonem, Ahmed F. Afify, Norazalina Saad, Yuki Shirosaki, Che Azurahanim Che Abdullah, Rania F. El Naggar, Mohammed A. Rohaim, Muhammad Munir

**Affiliations:** ^1^Department of Virology, Animal Health Research Institute, Agriculture Research Center (ARC), Giza, Egypt; ^2^Department of Physics, Faculty of Science, Universiti Putra Malaysia (UPM), Selangor Darul Ehsan, Malaysia; ^3^Laboratory of Cancer Research UPM-MAKNA (CANRES), Institute of Bioscience, Universiti Putra Malaysia, Selangor Darul Ehsan, Malaysia; ^4^Faculty of Engineering, Kyushu Institute of Technology, Kitakyushu, Japan; ^5^Research Center of Synthetic Biology, Kyushu Institute of Technology, Kitakyushu, Japan; ^6^Collaborative Research Centre for Green Materials on Environmental Technology, Kyushu Institute of Technology, Kitakyushu, Japan; ^7^Department of Virology, Faculty of Veterinary Medicine, University of Sadat City, Sadat City, Egypt; ^8^Department of Virology, Faculty of Veterinary Medicine, Cairo University, Giza, Egypt; ^9^Division of Biomedical and Life Sciences, Faculty of Health and Medicine, Lancaster University, Lancaster, United Kingdom

**Keywords:** cell culture techniques, viral contamination, EBV, OvHV-2, detection methods

## Abstract

Cell culture techniques are increasingly favored over animal models due to rising costs, time constraints, and ethical concerns regarding animal use. These techniques serve critical roles in disease modeling, drug screening, drug discovery, and toxicity analysis. Notably, cell cultures facilitate primary virus isolation, infectivity assays, biochemical studies, and vaccine production. However, viral contamination in cell cultures poses significant challenges, particularly due to the necessity for complex and sophisticated detection methods. Among the prevalent viruses, Epstein Barr virus (EBV) is ubiquitous across human populations, infecting approximately 98% of individuals. Despite its prevalence, the detection of EBV is often not considered a safety priority, as its detection methods are well-established, including PCR assays that can identify both active and latent forms of the virus. Conversely, ovine herpesvirus 2 (OvHV-2), a relative of EBV, presents a critical concern due to its ability to infect a wide range of organs and species, including over 33 animal species and nearly all domestic sheep. This makes the detection of OvHV-2 crucial for the safety of cell cultures across various species. The literature reveals a gap in the comprehensive understanding of both EBV and OvHv-2 detection in cell culture systems, highlighting an urgent need for developing robust detection methodologies specific to EBV and OvHv-2 to ensure bioprocess safety.

## 1 Introduction

Animal models have long been utilized in research by scientists. Zebrafish, mice, rats, rabbits, dogs, and primates such as the rhesus macaque have all been utilized as models over the years for diverse purposes and are still utilized in research today. Because humans and non-human animals have comparable genetic and physiological makeups and because diseases that affect animals can also affect humans, scientists around the world continue to employ animals to investigate human diseases. For instance, the pancreas of a dog was utilized to illustrate how it contributes to diabetes (Bédard et al., 2020). Another advantage of animal models is that they often have a shorter life expectancy than humans; therefore, scientists can study diseases over a lifetime in less time than it would take in a human body, such as studying metastasis (Schöffmann, 2011). Moreover, animal models should be replaced by more accurate and innovative *in vitro* models. Currently, some toxicity tests have failed to accurately predict human responses, highlighting the need for improved models (Stengelin et al., 2022). Economic considerations also encourage the shift toward alternative in vitro models. While cell culture techniques provide considerable economic, time, and ethical benefits, they are best used as complementary systems to animal models. Cell cultures enable high throughput testing and mechanistic research throughout the early stages of drug development. However, comprehending complex physiological connections and systemic impacts needs the continuous use of animal models in future research phases (Deschamps et al., 2022). However, the complexity of living organisms, including interactions across multiple organ systems, immunological responses, and pharmacokinetics, necessitates the continuous use of animal models to understand the broader physiological context of a drug’s effects (Deschamps et al., 2022). This strategy will improve the rigour of research by ensuring that findings from cell culture studies can be translated into more accurate predictions of *in vivo* results.

Ovine herpesvirus 2 (OvHV-2) is recognised as a substantial contaminant in cell cultures, posing potential challenges to research, particularly in the biotechnology and pharmaceutical industries. This virus primarily affects sheep, but it can infect a wide range of animal species, making it a concern for laboratories working with a diverse range of animal models. OvHV-2 is known to cause malignant catarrhal fever (MCF) in susceptible animals resulting in significant morbidity and mortality, especially in cattle and bison. Its presence in cell cultures, whether intentional or accidental, can lead to misinterpretations of experimental results, undermining the credibility of research findings. Therefore, detecting OvHV-2 contamination is critical for maintaining the integrity and accuracy of cell-based experiments (O’Toole and Li, 2014).

Epstein Barr virus (EBV) and ovine herpesvirus 2 (OvHV-2) are gammaherpesviruses with high prevalence and worldwide distribution (Hart et al., 2007; Bastawecy et al., 2023). Therefore, the presence of their latent and active forms can be problematic for human and animal cell technology used in the production of biologicals for prophylaxis and therapy (Tonoyan, et al., 2024). Our study sheds light on the rapid screening of EBV and OvHV-2 contamination in cell cultures of humans, animals, or insects in cell banks, ensuring quality and safety through continuous and daily inspections.

## 2 Challenges and alternatives in animal-based pharmacological studies

The increasing costs, time, and ethical concerns about animal use are significant issues. *In vitro* pharmacological studies of nano delivery can mimic the *in vivo* system. This provides a straightforward method to investigate the effects of such materials without endangering animals, especially during the screening phase. Stress exposure in the form of nutrient deprivation or drug-induced toxicity could lead to necrotic or apoptotic death at the cellular level (Kura et al., 2014). The significant challenges faced by modern-day medicine include designing a target-specific drug delivery system with a controlled release mechanism, having the potential to avoid opsonisation and reduce bio-toxicity. Nanoparticles, which may be naturally occurring or synthetically engineered, are materials characterized by their nanoscale dimensions. Engineered nano-sized materials are playing an indispensable role in the fields of nanomedicine and nanobiotechnology (Taghipour et al., 2018). 2D and 3D mammalian cell-based assays are widely used to model diseases, screen drugs, discover drugs, and analyse toxicity (Rachamalla et al., 2021).

Cell cultures have become useful tools in pharmacological research, particularly in early-stage drug discovery, due to their ability to provide cost-effective and rapid screening systems (Weiskirchen et al., 2024). The capacity to test drugs in cell cultures allows for the research of specific pathways, receptor interactions, and metabolic processes, all of which are critical for understanding therapeutic efficacy and safety (Weiskirchen et al., 2024). However, while cell cultures can substantially reduce the reliance on animal models during early research stages, they do not fully replicate the complexities of whole-body physiology, including the influence of the immune system, metabolic processes, and multi-organ interactions. As such, the indispensable role of animal studies remains in later phases of research, particularly for assessing systemic effects, long-term toxicity, and therapeutic efficacy in a living organism (Ejma-Multański et al., 2023). Thus, while cell cultures provide invaluable insights in the early phases, animal models continue to be essential for translating these findings into viable, safe, and effective treatments.

The ultimate source of cells for cell culture is the intact animal. Cells may be obtained from various organs and tissues of embryonic, infant, or adult origin. Cultures of animal cells are usually divided into three classes: primary cells, cell strains, and cell lines, as depicted in Table 1. This advancement is particularly relevant when considering the challenges posed by viral contamination, such as that from Epstein–Barr virus (EBV) and ovine herpesvirus 2 (OvHV-2), which can significantly impact experimental outcomes (Table 2).

**Table 1:** Characteristics of primary cells, cell strains, and cell lines.

	Primary cells	Cell strains	Cell lines
Source	Derived directly from tissues of an organism (e.g., human, animal, plant)	Derived from primary cells that have been sub-cultured and adapted to *in vitro* conditions, often by selection or genetic modification	Derived either from primary cells that have undergone transformation (spontaneously or artificially induced) or from cancerous tissues
Lifespan	Finite; can only undergo a limited number of cell divisions before senescence, typically around 5–10 divisions, though this varies depending on the cell type	Still finite but usually longer than primary cells; typically, can undergo more divisions, sometimes up to 100 divisions before senescence	Immortal; they can divide indefinitely in culture as long as appropriate conditions are maintained
Karyotype	Retain their original diploid chromosome number and genetic characteristics similar to their tissue of origin	May retain a diploid chromosome number, but there can be slight changes due to adaptation to in vitro conditions	Often aneuploid, with significant alterations in chromosome number and structure due to the transformation process
Growth properties	Exhibit normal growth characteristics, closely mimicking the *in vivo* environment. They generally require specialized conditions and media for growth	Can have more stable and consistent growth characteristics compared to primary cells. However, they may start to diverge somewhat from the original tissue characteristics over time	Tend to have robust and rapid growth, often requiring less specialized conditions compared to primary cells or cell strains
Heterogeneity	Often heterogeneous, consisting of multiple cell types similar to those found in the original tissue	More homogeneous than primary cells, often consisting of a more uniform population of cells due to the selection process	Generally, more homogeneous than primary cells, though some variability can occur, especially in long-term cultures
Usage	Ideal for studying normal physiology, cellular functions, and responses to stimuli, but limited by their short lifespan and difficulty in long-term maintenance	Useful for research that requires a longer-term culture with relatively consistent characteristics, such as drug testing or genetic studies	Widely used in research, especially in studies requiring large quantities of cells, genetic studies, cancer research, and drug development. However, their altered properties can limit their applicability to studies of normal cellular physiology

**Table 2:** Susceptible cell lines and preferred detection methods for Epstein–Barr virus (EBV) and ovine herpesvirus 2 (OvHV-2) contamination.

Virus	Cell line	Description	Preferred detection methods	References
EBV	B-lymphoblastoid cell lines (B-LCLs)	Immortalized human B-cell lines widely used in immunology and genetic studies	PCR: Detects EBV DNA with high sensitivity and specificity *In situ* hybridization (ISH): Detects EBV-encoded small RNAs (EBERs) EBNA detection: Identifies EBNA proteins via ELISA or Western blot	Hess (2004), Sun et al. (2024), and Liu et al. (2021)
	293 human embryonic kidney (293HEK)	Immortalized human kidney cells commonly used in cell biology and virology	PCR: Rapid detection of EBV DNA Southern blot: Differentiates between latent (episomal) and lytic (linear) EBV DNA Western blot: Detects ZEBRA and other lytic cycle proteins	Hess (2004) and Uphoff et al. (2010)
	B95-8 marmoset lymphoblastoid cells	Known producer of infectious EBV particles, used in viral propagation studies	EBV antigen detection: Identifies EA, VCA, and EBNA using specific antibodies Electron microscopy: Visualizes EBV particles, particularly during lytic infection	Castro and Heuschele (1992) and Sun et al. (2024)
OvHV-2	Ovine peripheral blood lymphocytes	Primary target cells for OvHV-2 in sheep, important for studying viral pathogenesis	PCR: Main tool for detecting OvHV-2 DNA Quantitative PCR (qPCR): Precisely quantifies viral load *In situ* hybridization: Detects viral RNA, confirming active infection	Phillips et al. (2018) and Wiyono et al. (2023)
	Bovine and deer lymphocyte cultures	Cultures used to study cross-species transmission of OvHV-2	PCR: Standard detection method for OvHV-2 DNA Immunofluorescence assay (IFA): Detects viral antigens Western blot: Confirms viral protein presence	Phillips et al. (2018) and Bastawecy et al. (2023)
	Madin-Darby bovine kidney (MDBK)	Used in research on OvHV-2 replication and pathogenesis, though less commonly infected	PCR: Effective for detecting low levels of OvHV-2 DNA Western blot: Confirms viral protein expression during active infection	Castro and Heuschele (1992) and Phillips et al. (2018)

In the realm of virus culture, high-throughput screening platforms have become essential tools for drug discovery. These platforms facilitate the rapid evaluation of compounds for antiviral activity, thereby streamlining the process of identifying effective treatments (Zhu et al., 2021). Advanced 3D models integrated into drug testing further enhance our ability to simulate the tissue microenvironment, which is critical for assessing drug responses and side effects. By incorporating patient-derived cell lines and organoids, researchers can gain insights into individual variations in drug efficacy and toxicity, providing a more personalised approach to drug development (Baker, 2021).

Despite these technological advancements, the issue of cell line contamination with viruses like EBV and OvHV-2 persists, posing significant challenges for research. To mitigate these risks, robust quality control measures, including short tandem repeat (STR) profiling and mycoplasma testing, have proven to be effective in ensuring the authenticity and integrity of cell cultures (Marsh et al., 2019). These measures are particularly important for maintaining the reliability of experiments and protecting the safety of therapeutic applications. In conclusion, while modern cell culture technologies offer powerful tools for studying viruses and testing drugs, integrating these advancements with animal models remains crucial for validating research findings and ensuring their applicability to real-world scenarios. By addressing viral contamination concerns and leveraging the latest technological innovations, we can enhance the reliability and safety of biotechnological research (Sun et al., 2024).

## 3 Challenges of viral contamination in cell cultures

Unlike microbial contamination, which is typically straightforward to detect, viral contamination presents significant challenges due to the difficulty in identifying some viruses and the lack of effective treatment options for infected cultures. However, some viruses induce distinct cytopathic effects that can be observed under a microscope. In the study of viral infections, observing and documenting the cytopathic effects (CPE) induced by various viruses in cell cultures is crucial for understanding the impact of these pathogens on host cells. CPE refers to the visible alterations in cell morphology caused by viral replication and is an important diagnostic feature in virology (Leland and Ginocchio, 2007). These effects can include cell rounding, syncytia formation, and cell lysis, and they vary depending on the virus and the cell line used (Leland and Ginocchio, 2007).

**Figure 1:**
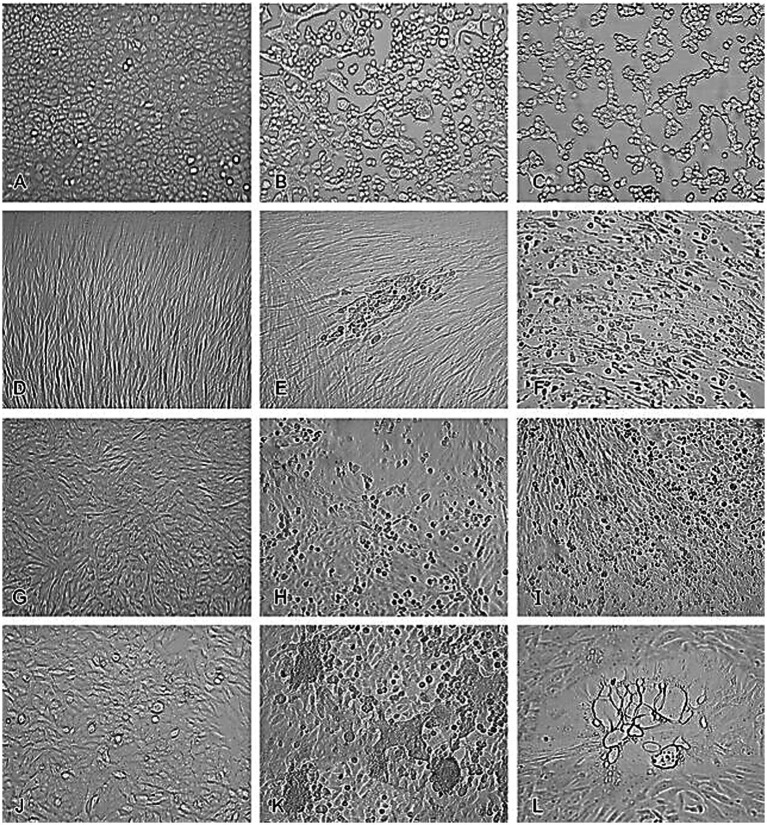
Cytopathic effects of common viruses in various cell lines. Uninfected cell cultures and cell cultures showing viruses-induced CPE. **(A)** Uninfected A549 cells. **(B)** HSV-2 in A549 cells. **(C)** Adenovirus in A549 cells. **(D)** Uninfected MRC-5 fibroblasts. **(E)** CMV in MRC-5 fibroblasts. **(F)** Rhinovirus in MRC-5 fibroblasts. **(G)** Uninfected RhMK cells. **(H)** Enterovirus in RhMk cells. **(I)** Influenza A virus in RhMk cells. **(J)** Uninfected HEp-2 cells. **(K)** RSV in HEp-2 cells. **(L)** Monkey virus contaminant in RhMk cells (adapted from Leland and Ginocchio (2007) with permission).

For instance, uninfected A549 cells (Figure 1A) typically exhibit a uniform and healthy appearance, but when infected with HSV-2, these cells undergo significant morphological changes, including rounding and detachment from the culture surface (Figure 1B). Similarly, adenovirus infection in A549 cells leads to cell aggregation and granulation, clearly visible through microscopic examination (Figure 1C) (Leland and Ginocchio, 2007). In MRC-5 fibroblasts, uninfected cells display normal fibroblast morphology (Figure 1D), but infection with CMV results in the formation of enlarged cells containing characteristic intranclear inclusions (Figure 1E). Rhinovirus infection in the same cell line causes cytoplasmic vacuolation (Figure 1F), further demonstrating the range of morphological changes viruses can induce, compared to uninfected RhMK cells (Figure 1G) (Leland and Ginocchio, 2007). RhMK cells are commonly used for isolating respiratory viruses, show dramatic CPE when infected with the enterovirus, including cell rounding and clumping (Figure 1H). Infection with influenza A virus in RhMK cells leads to similar morphological disruptions (Figure 1I) (Leland and Ginocchio, 2007). Moreover, Compared to uninfected HEp-2 cells (Figure 1J), RSV infection in HEp-2 cells causes the formation of syncytia (Figure 1K), where multiple cells fuse to form large, multinucleated cells. Finally, monkey virus contamination in RhMK cells can be identified by the presence of vacuolated cells, indicating substantial cellular disruption (Figure 1L) (Leland and Ginocchio, 2007).

In contrast, some viral infections result in the integration of viral DNA into the host genome, forming a provirus without altering the cell’s morphology (Skalka and Katz, 2005). This type of infection can be challenging to detect since it does not produce visible changes, posing risks to other cell lines and potentially affecting researchers and patients, particularly in the production of injectable biological products (Meyer et al., 2017).

## 4 Implications of EBV contamination in cell cultures

Viral infections can originate from contaminated cell lines, contaminated raw materials, or from breakdowns in the production and purification processes. All cell lines established using viral transformation, such as EBV-transformed B lymphocytes, have the potential to produce the virus used for transformation (Abdelmonem et al., 2024). Therefore, they also represent a potential infection risk to operators, the cell culture lab, and patients receiving biologicals produced with such cell lines. In general, viral contaminations of cell lines cannot be treated, and contaminated cultures should be discarded (Merten, 2002).

**Figure 2:**
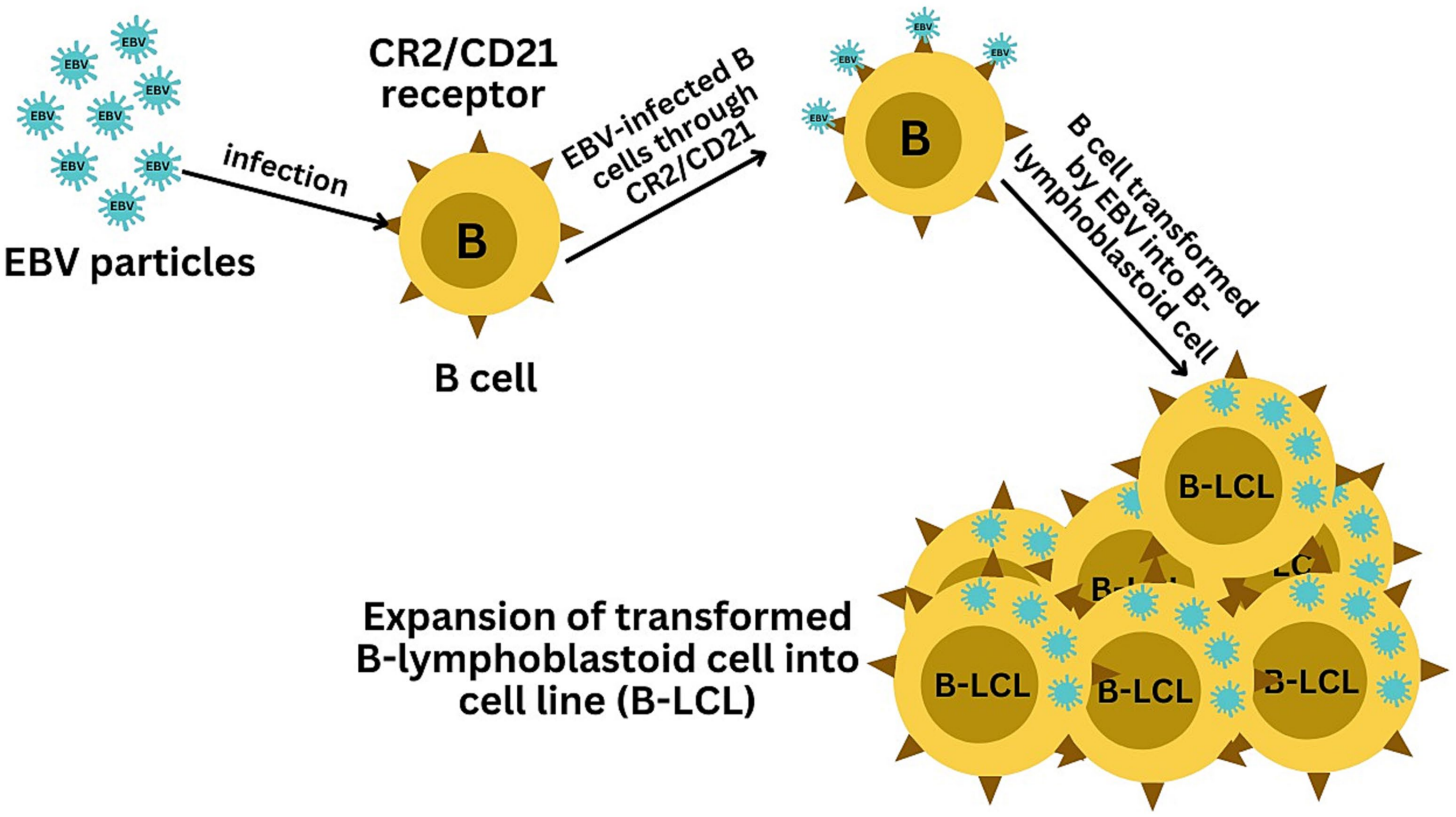
Illustration of the EBV-mediated transformation of B cells into B-lymphoblastoid cell lines (B-LCLs). The infection starts with EBV binding to B cells via the CR2/CD21 receptor, initiating viral entry. The final panel depicts the resulting B-LCLs, which are characterized by their sustained growth and the presence of EBV DNA, demonstrating the successful transformation and immortalization of the initially infected B cells.

**Figure 3:**
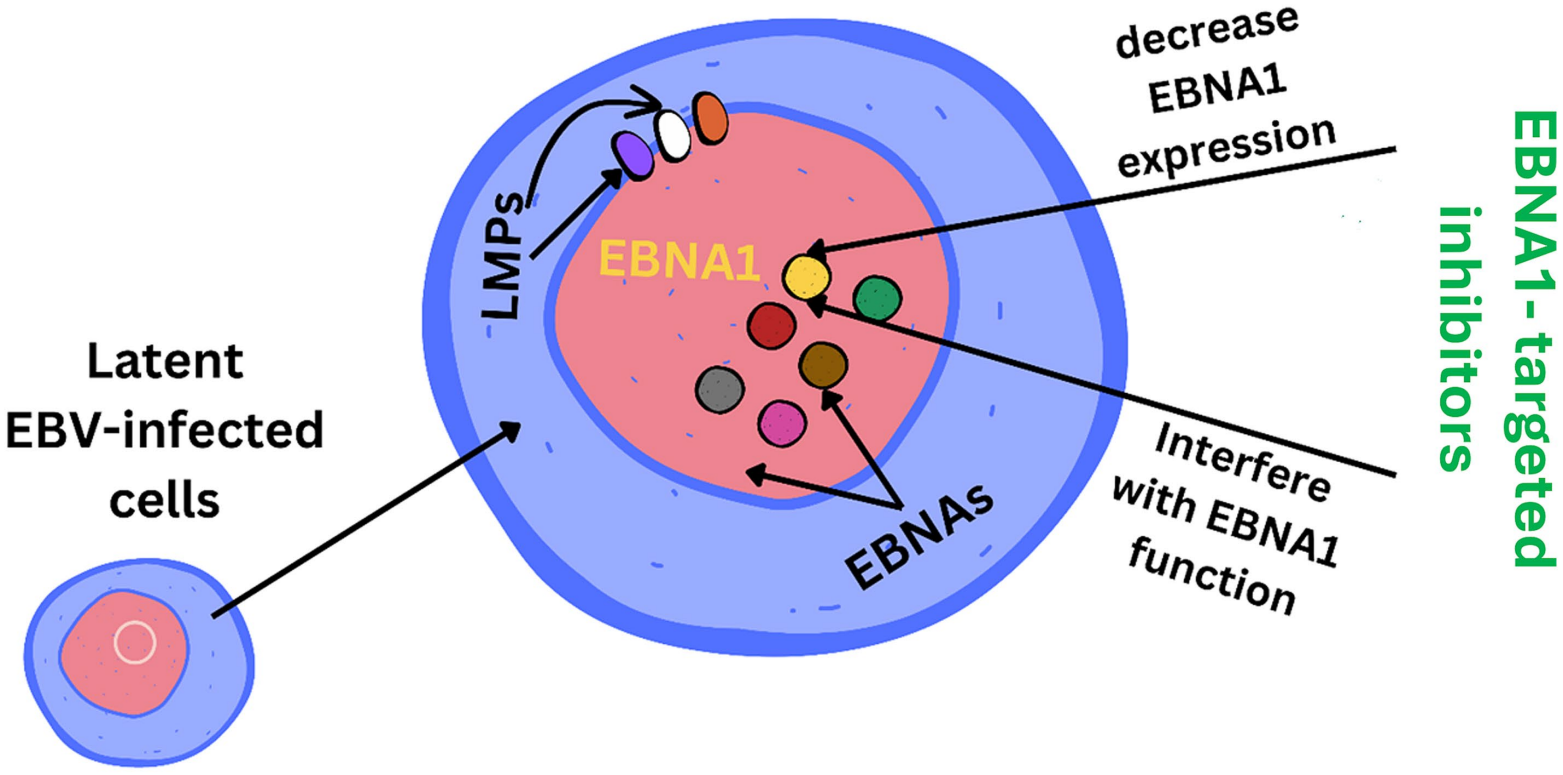
EBNA1 (Epstein–Barr nuclear antigen 1) is a vital Epstein–Barr virus (EBV) protein responsible for tethering the viral genome to host chromosomes, ensuring its persistence in dividing cells. Inhibitors targeting EBNA1 are crucial for maintaining the viral genome and its associated oncogenic potential in EBV-infected cells.

**Figure 4:**
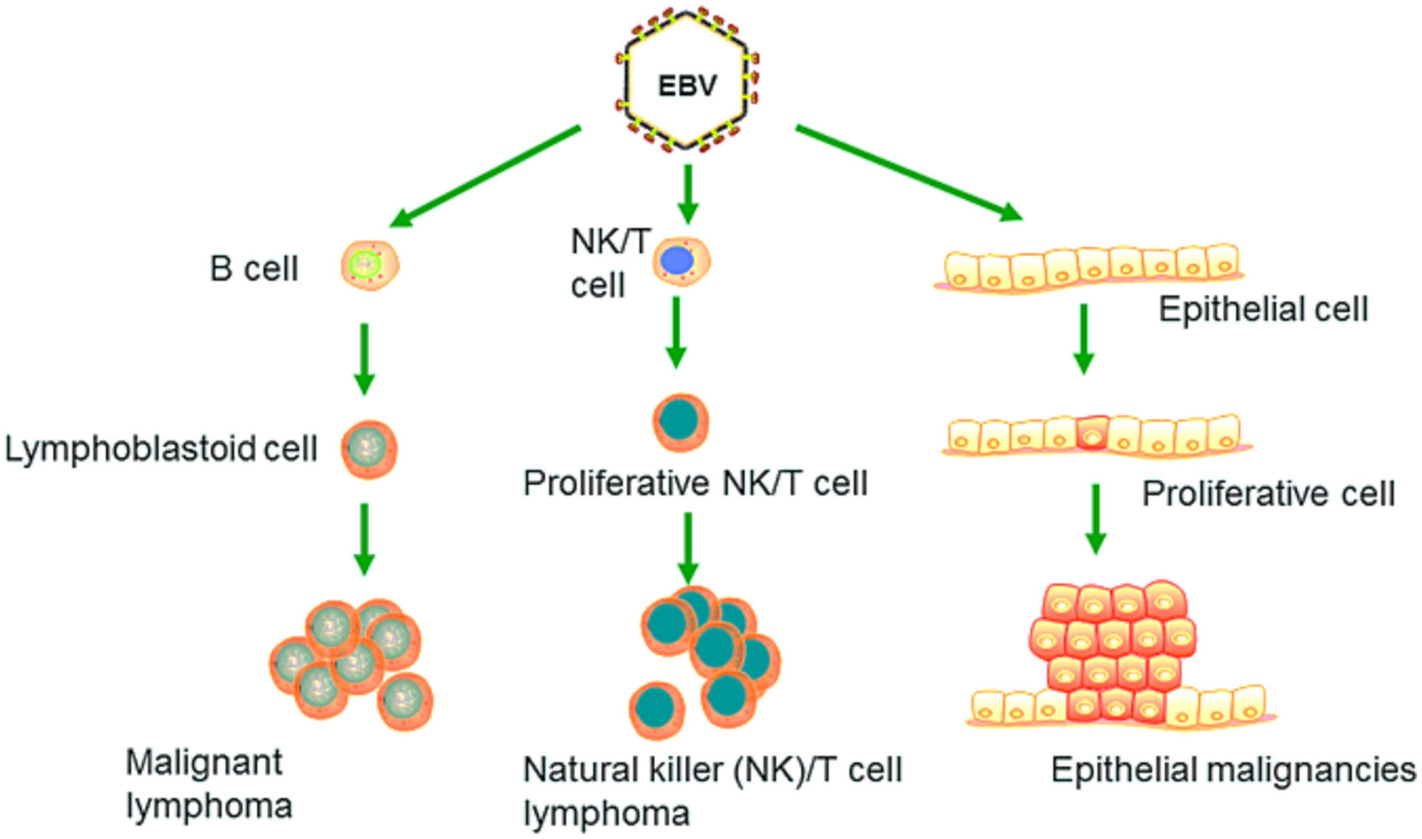
EBV primarily targets B lymphocytes, leading to their malignant transformation and the development of cancers such as Burkitt lymphoma. It can also induce malignant changes in epithelial cells, resulting in cancers such as nasopharyngeal carcinoma and EBV-associated gastric carcinoma. Recent research has revealed that EBV is capable of infecting NK and T cells, which can lead to the formation of natural killer and T cell lymphoma (Adapted from Yin et al. (2019) with permission).

EBV has become a prime example of a human tumor virus that is etiologically linked to a diverse range of malignancies. Additionally, EBV uniquely has the capability to transform, and immortalize resting B cells into permanently growing B-lymphoblastoid cell lines (B-LCL) as illustrated in Figure 2. EBV is ubiquitously distributed in all human populations, with approximately 98% of individuals infected (Smatti et al., 2017). Therefore, the detection of EBV is not primarily a matter of safety. However, for quality control reasons and due to the EBV’s potential to transform B cells, cell banks should routinely determine the EBV infection status of cell lines. Not every cell line derived from a tumor patient is necessarily a tumor cell line, as non-malignant cells that are independent of the tumor cells may also be immortalized. In a leukemia context, such cell lines are usually normal B cells which become immortalized through the incorporation of the EBV genome (Uphoff et al., 2010). However, EBV was declared a class 1 carcinogen by the International Agency for Research on Cancer and the World Health Organization in the late 1990s. EBV displays prolonged latency in lymphocytes, interfering with immune functions and potentially inducing cell proliferation and transformations. EBV infection involves many organ systems and is often misdiagnosed or underdiagnosed. Therefore, early diagnosis and rational treatment are extremely important. During latent EBV infection, the virus is detectable in the nucleus in a ring form, linked to the chromatin of the host genome by the Epstein–Barr viral nuclear antigen 1 (EBNA1) protein (Sun et al., 2024). The EBNA-1 efficiently tethers the viral DNA to the host chromosome, which is duplicated throughout mitosis and provided to both daughter cells (Hu et al., 2016). Thus, EBV remains mostly latent in an infected organism. Due to EBNA-1’s functions in the maintenance, replication, and segregation of the EBV genome, it can be attractive for designing specific EBNA-1 inhibitors (Figure 3), to decrease EBNA-1 expression or interfere with EBNA-1-dependent functions (Jiang et al., 2018). EBV transforms human B-lymphocytes into proliferating blasts, which can be efficiently established into continuous cell lines. Such cell lines contain the viral DNA as a nuclear plasmid (Kriegler, 1990). Since EBV has a strong tropism for B lymphocytes and the capacity to activate them to proliferate continuously (Hatton et al., 2014; Frappier, 2021) when epithelial cells (Figure 4), are exposed to *in vitro* infection-free cells, we observe a very low level of infection; however, when these cells are associated with infected B cells, the levels of infection increase significantly (Sangueza-Acosta and Sandoval-Romero, 2018).

## 5 Advanced detection techniques for EBV in cell cultures

Epstein–Barr virus (EBV) is classified under the genus *Lymphocryptovirus* in the *Herpesviridae* family and belongs to the *gammaherpesvirinae* subfamily. This virus is associated with conditions such as infectious mononucleosis and several malignancies, including Burkitt lymphoma and nasopharyngeal carcinoma (Young and Rickinson, 2004). The EBV genome is a double-stranded DNA molecule approximately 172 kilobases long, encoding a range of latent and lytic proteins such as Epstein–Barr nuclear antigens (EBNA) and latent membrane proteins (LMP), which are crucial for the virus’s ability to transform and persist within host cells (Damania et al., 2022).

Multiple reports have described the detection of EBV infection in various cell types maintained in cell culture banks (Uphoff et al., 2010; Edwards et al., 2019; Suzuki et al., 2024; Tomomasa et al., 2024). These infections can be attributed to the presence of an existing EBV infection during the initial process of cell line establishment, EBV contamination of culturing materials, or improper manipulation by experimental staff. *In situ* hybridization assays, which detect EB early RNA—a small EBV-encoded RNA continuously transcribed and expressed after infection—are the current gold standard for detecting EBV infection in clinical settings (Uphoff et al., 2010; Nakatsuka et al., 2015). However, this technique is applicable only to tissue samples. Nonetheless, a simple system that allows for the rapid detection of EBV in multiple contexts, including both cell culture and tissue samples, remains necessary (Sun et al., 2024).

**Figure 5:**
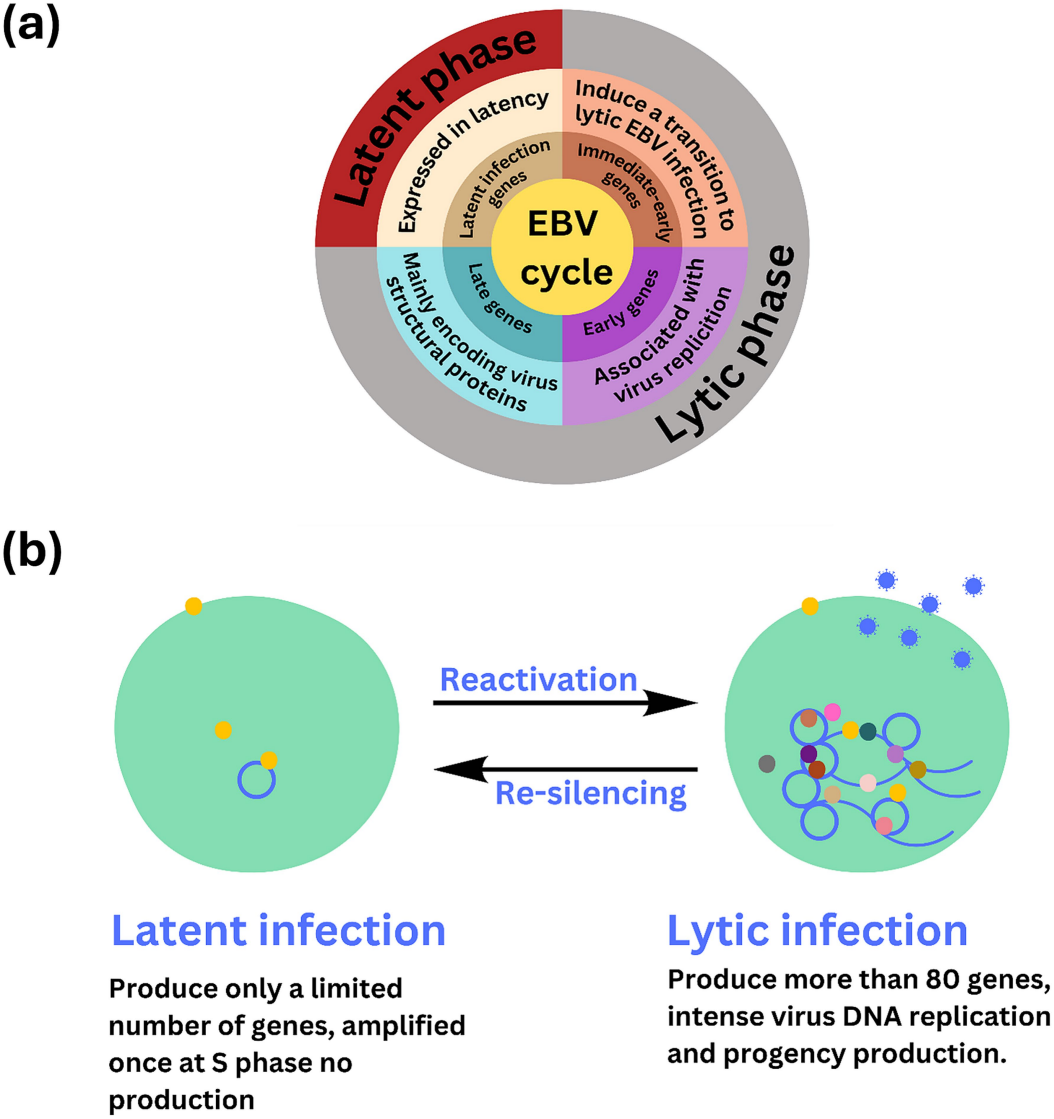
Overview of stable, non-replicative latent phase and the active, replicative lytic phase of EBV including the regulatory mechanisms and gene expression changes involved in each stage. **(A)** The Epstein–Barr virus (EBV) lifecycle is characterized by two distinct phases: latent and lytic. During the latent phase, the virus remains dormant within infected cells, primarily residing in **B** lymphocytes. In this state, EBV maintains at low level of gene expression and replication. Key latent proteins, including EBNA1, EBNA2, and LMPs (latent membrane proteins), are expressed, allowing the virus to persist in the host without causing immediate cell death. This phase is crucial for the long-term maintenance of the virus within the host, contributing to chronic infections and potential oncogenic processes. **(B)** EBV infection has two possible states, latent and lytic. The yellow circles indicate viral proteins involved in viral latency, and the gray, brown, light and dark pink, dark green, and yellow circles indicate the viral proteins involved in lytic infection and release of EBV (blue circle). The active state of the virus may sometimes be “re-silencing,” which may contribute to oncogenesis.

Long non-coding RNAs act by regulating target genes and are involved in tumorigenesis. EBV genes, can be divided into latent infection genes, immediate-early genes, early genes, and latent genes, depending on when they are produced within the viral cycle (Liu et al., 2021) as shown in Figure 5A. Meanwhile, antigen detection is the easiest and most inexpensive method for testing, with commercial monoclonal antibodies to EBNA (1–6) available. Virus-specific antigens produced during an infection can be identified in experimentally infected cells that are fixed in various ways, to demonstrate the presence of the relevant antibody. The antigens shown in Table 3 are early antigen (EA), viral capsid antigen (VCA), nuclear (tumor) antigens (EBNAs), and membrane antigens (Hess, 2004). Several cell lines have been examined for EBV infection either by the originators of the cell lines or subsequently by other investigators. In the majority of cases, the EBV status of the cell lines was determined immunologically by detecting EBNA, EA, and/or VCA. The monkey cell line B95-8 is known to produce infectious EBV particles (Müller et al., 2001). However, only cell cultures producing active viruses should be considered to represent an elevated risk. To identify the lytic phase of EBV infections, expression of ZEBRA protein (BamHI Z Epstein–Barr replication activator) was analyzed by western blotting using an anti-ZEBRA monoclonal antibody (Münz, 2019; Cao et al., 2021). ZEBRA, the product of the BZLF1 gene, is a transcriptional activator that mediates a genetic switch between the latent and lytic states of EBV, as illustrated in Figure 5B. It binds to the promoters of genes involved in lytic DNA replication, activating their transcription (Wu et al., 2024).

**Table 3:** Summary of key Epstein–Barr virus (EBV) antigens and their roles in the viral lifecycle.

Antigen type	Antigen	Description	Role	References
Early antigens	EA-D (early antigen diffuse)	Component of the early antigen complex; found throughout the nucleus of infected cells	Associated with early stages of EBV replication	Kieff and Rickinson (2007)
	EA-R (early antigen restrictive)	Specific and localized early antigen; found in the cytoplasm	Involved in regulating viral replication	Kieff and Rickinson (2007)
Nuclear antigens	EBNA1 (Epstein–Barr nuclear antigen 1)	Binds to viral DNA and tethers it to host chromosomes; crucial for maintaining the viral episome	Ensures viral genome persistence during cell division	Young and Rickinson (2004)
	EBNA2 (Epstein–Barr nuclear antigen 2)	Activates viral and cellular genes; involved in B cell transformation	Essential for B cell proliferation and EBV-associated malignancies	Young and Rickinson (2004)
	EBNA3 (EBNA3A, EBNA3B, EBNA3C)	Modulates host cell gene expression and immune response; controls cell cycle	Contributes to immune evasion and viral latency.	Longnecker and Rowe (2010)
	LMP1 (latent membrane protein 1)	Mimics an active receptor; influences cellular signaling pathways.	Promotes cell survival and proliferation, involved in transformation	Young and Rickinson (2004)
	LMP2 (latent membrane protein 2)	Exists as LMP2A and LMP2B isoforms; modulates B cell receptor signaling	Maintains latent infection and regulates B cell signaling	Young and Rickinson (2004)
Membrane antigens	LMP1 (latent membrane protein 1)	(Also listed under nuclear antigens)	Acts as a constitutively active receptor, essential for cell transformation	Young and Rickinson (2004)
	LMP2 (latent membrane protein 2)	(Also listed under nuclear antigens)	Modulates B cell receptor signaling and latency	Young and Rickinson (2004)
Capsid antigens	VCA (viral capsid antigen)	Includes major capsid proteins (VCA-p18, VCA-p23); essential for capsid structure	Marker for active EBV infection and capsid formation	Kieff and Rickinson (2007)
	gp350/220 (glycoprotein 350/220)	Involved in the attachment of the virus to CD21 on B cells	Crucial for initial infection of new cells	Kieff and Rickinson (2007)

Not all latently infected lymphoblast cell lines can be induced by TPA. For example, marmoset lymphoblasts, such as the B95-8 cell line, appear to be more inducible. Therefore, the B95-8 cell line is used as a positive control to determine the efficiency of induction with TPA/NA-butyrate. The EBV genome can be present in host cells as covalently closed circular episomes, as linear DNA of active viruses, or integrated into the host genome. The episomes indicate a latent infection status. EBV-infected cells can harbor 1–10 episomes in low-load cells or up to several hundred episomes in high-load cells. EBV producer cell lines also contain linear double-stranded DNA, which is packaged into virions. To distinguish between the linear DNA of active viruses, the episomal DNA of EBV-infected cell cultures, and solely integrated EBV genomes, varieties of southern blot analysis were used (Uphoff et al., 2010).

Maintaining the quality of cellular products in biological research is critically important, particularly because Epstein–Barr virus (EBV) is prevalent and can contaminate cell lines (Uphoff et al., 2010). As a latent virus, EBV can spread between cell lines in laboratory settings, potentially compromising experimental results and safety (Uphoff et al., 2010). To effectively address this issue, several advanced molecular detection methods are utilized. PCR-based detection remains fundamental due to its high sensitivity and ability to amplify viral DNA, making it essential for identifying both latent and active EBV contamination in cell lines (Uphoff et al., 2010). Complementing this, recombinase polymerase amplification (RPA) offers rapid and efficient detection by amplifying DNA at a constant temperature, with the RPA-lateral flow assay (LFA) providing a straightforward visual method for screening EBV in various samples (Sun et al., 2024).

Additionally, *in situ* hybridisation detects early EBV RNA continuously expressed post-infection, and while primarily used for tissue samples, it sets a high standard for EBV detection in clinical environments (Uphoff et al., 2010). Immunoassays such as ELISA and Western blotting further support EBV detection by identifying specific viral antigens and proteins such as early antigen (EA) and viral capsid antigen (VCA), crucial for understanding the infection status (Hess, 2004). For more detailed analysis, mass spectrometry characterises EBV proteins and metabolites, enhancing our understanding of virus-host interactions (Cao et al., 2021). Electron microscopy provides a visual dimension by enabling the observation of EBV particles and cellular ultrastructure, which aids in understanding viral morphology and infection mechanisms (Machón et al., 2019). Finally, Southern blot analysis helps in distinguishing between integrated EBV genomes, episomal DNA, and linear viral DNA. This differentiation is essential for identifying latent versus active infections and gaining insights into the viral life cycle (Gulley and Tang, 2008; Uphoff et al., 2010). By integrating these methods, researchers can comprehensively monitor and detect EBV contamination, with each technique contributing to ensuring cell line integrity and experimental safety (Berthomé et al., 2010; Sun et al., 2024).

## 6 Risk of OvHV-2 contamination in cell cultures

**Figure 6:**
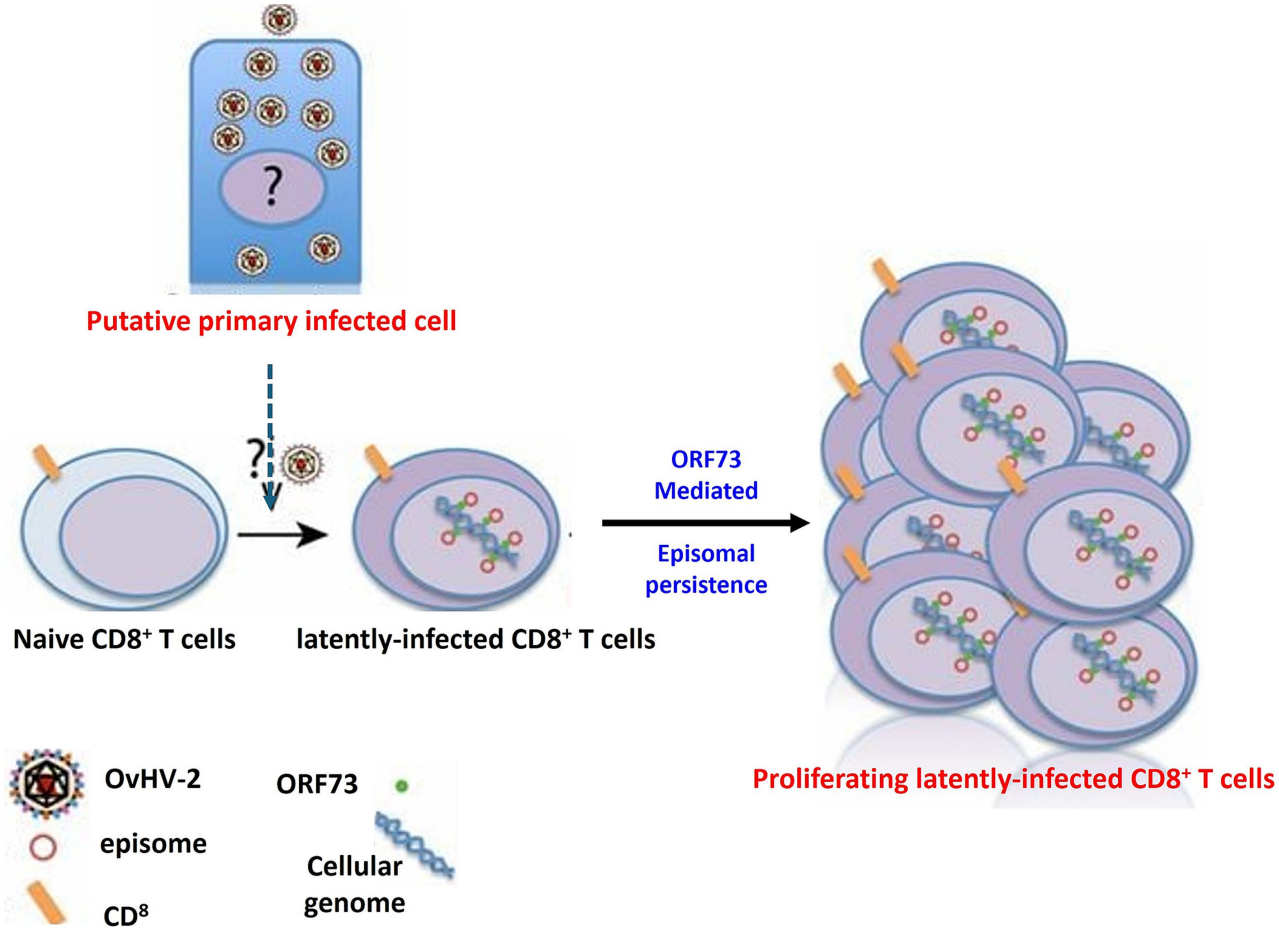
The mechanism of OvHV-2 latency involves the establishment and persistence of the virus within CD8^+^ T cells. This process likely begins with a primary productive infection in as-yet-unknown cell types, after which CD8^+^ T cells become infected. In these cells, OvHV-2 establishes latency by maintaining viral episomes within the nucleus, potentially through the expression of proteins similar to ORF73 leading to the proliferation of CD8^+^ T cells.

Ovine herpesvirus 2 (OvHV-2) poses a significant challenge in research settings, notably in biotechnology and pharmaceutical facilities, due to its ability to contaminate cell cultures and alter experimental results (O’Toole and Li, 2014). One of the most concerning aspects of OvHV-2 is its ability to establish latency (Figure 6), which hampers detection and interpretation of experimental results. During latency, the virus remains in host cells without causing active viral replication, frequently escaping the immune response (Dry et al., 2019). This silent presence can be especially problematic in cell culture systems, where the virus may not induce visible cytopathic effects but nevertheless influence cell behavior, immunological responses, and experimental outcomes. Because OvHV-2 can enter and maintain latency, researchers must be diligent in screening for its presence, as even subclinical or latent infections might result in biased results, undermining the validity of cell-based research. As a result, knowing the molecular mechanisms underlying OvHV-2 latency is crucial for creating effective detection methods and verifying the accuracy of research findings, particularly in studies that rely on cell cultures from various animal models.

OvHV-2 shares many characteristics with EBV, as both belong to the *Herpesviridae* family and the *gammaherpesvirinae* subfamily. OvHV-2 is the primary cause of malignant catarrhal fever worldwide and affects virtually all domestic sheep, with both domestic and wild sheep serving as natural reservoirs (Bastawecy et al., 2023; Phillips et al., 2018). This virus can impact almost any organ, as indicated by its broad range of clinical manifestations (Wiyono et al., 2023). The OvHV-2 genome is a double-stranded DNA molecule approximately 138 kilobases in length, encoding proteins similar to those of EBV, which are involved in immune evasion and cellular transformation (AlHajri et al., 2017; Moré et al., 2024). For isolation and characterization, foetal ovine kidney cell cultures have been used to identify herpesvirus particles through electron microscopy, revealing intranuclear vesicles (Castro and Heuschele, 1992). Furthermore, OvHV-2 can be isolated using embryonated chicken eggs, via both the yolk sac and chorioallantoic membrane routes (Constable et al., 2016). The need for comprehensive screening is emphasized by the fact that herpesvirus can also be present in a wide range of specimens, including lung, kidney, liver, and other tissues (Castro and Heuschele, 1992). Thus, it is essential to screen cell lines from all species for OvHV-2 to prevent contamination and ensure the reliability of research and biotechnology applications.

## 7 Transplacental transmission risks of EBV and OvHV-2 in cell cultures

Transplacental transmission is a possible route for EBV transmission (Kim et al., 2017), and similar dissemination of OvHV-2 has been detected in an asymptomatic calf. Although this calf contained viral DNA, it showed no clinical manifestations of MCF (O’Toole et al., 1997). Additionally, vertical transmission of OvHV-2 was inferred in virus-free and gnotobiotic lambs (Headley et al., 2015; Headley et al., 2020; Rosato et al., 2021). Moreover, OvHV-2 was identified within multiple tissues of a cow and its 4-month-old fetus, demonstrating the transplacental transmission of OvHV-2 in SA-MCF in cattle (Headley et al., 2015). Therefore, cell lines obtained from fetuses or newborns must be screened for EBV and OvHV-2. Insects may also be infected with EBV or OvHV-2, as evidenced by some patients who were detected to have hypersensitivity to mosquito bites associated with chronic EBV infection (Chiu et al., 2016). Additionally, vectors, including insects, could transmit OvHV-2 (Li et al., 2001). OvHV-2 causes MCF, which occurs in both acute and chronic forms and has a morbidity rate that usually varies from 15 to 100% in cattle (Zakharova et al., 2020). MCF has been reported in more than 33 species, including cattle, buffalos, deer, giraffes, pigs, sheep (Wiyono et al., 2023), goats (Makoni et al., 2024), equines (Costa et al., 2009; Madrigal-Valencia et al., 2023), New World camelids such as alpacas (Goerigk and Merbach, 2012), and Old World camelids, such as camels (Hristov and Peshev, 2016). Experimental animals, such as guinea pigs (Constable et al., 2016), rabbits, and hamsters, are also susceptible to this virus (Russell et al., 2009). The absence of the virus can only be assured by performing a rigorous testing program, which includes all steps in a bioprocess: master cell bank, working cell bank, raw materials, unprocessed bulk harvest, late expanded cells, and the final product (Merten, 2002). These approaches must be applied to EBV, which is known for its high prevalence (Smatti et al., 2017) and OvHV-2, which is also known to be endemic in most sheep and goat populations (Yildirim et al., 2012).

## 8 Conclusion

EBV and OvHV-2 represent significant threats to the integrity and safety of cell cultures in biomedical research and bioproduction. Both viruses, notorious for their latent infection capabilities, complicate detection efforts and increase the risk of contamination in cell culture banks. EBV, a class 1 carcinogen associated with various malignancies, and OvHV-2, linked to malignant catarrhal fever with high morbidity in diverse animal species, underscore the necessity for stringent testing and vigilant monitoring in both research and therapeutic applications.

Given the potential for transplacental and cross-species transmission of these viruses, it is imperative that newly established cell lines, whether human or animal-derived, undergo rigorous testing for EBV and OvHV-2. The detection of EBV-transformed B-cells, which can give rise to B-LCLs, is particularly crucial, as these cells can skew research outcomes and compromise biotherapeutic production. Advanced diagnostic techniques, such as sequencing the B glycoprotein gene of EBV and comparing it with that of OvHV-2, should be employed to enhance detection accuracy and prevent false results.

## 9 Future perspectives and practical applications

To protect the quality of cell cultures, it is crucial to use a comprehensive strategy that combines advanced detection methods with rigorous lab practices. Looking ahead, developing highly sensitive and precise diagnostic tools, potentially utilizing CRISPR-based technologies, could offer innovative solutions for the continuous monitoring of cell lines in research settings. Alongside improvements in diagnostics, it is essential to enforce strong quality control measures to prevent contamination by EBV and OvHV-2. This includes regular cell line screenings and adherence to good laboratory practices (GLP). Such practices are critical not only for research but also for the pharmaceutical and biotechnology industries, where cell line purity is crucial for producing biologics and vaccines. Regular screening for EBV and OvHV-2 during production can avoid expensive contamination issues and ensure the safety of therapeutic products.

As the field progresses, incorporating high-throughput sequencing and single-cell analysis into EBV and OvHV-2 detection processes could reveal viral diversity within cell cultures. This could lead to the identification of new viral variants, guide targeted interventions, and provide deeper insights into viral latency and reactivation. Additionally, establishing a standardized protocol for screening all cell cultures for OvHV-2 before use is strongly recommended. Such preventative steps are necessary to preserve the biological integrity of cell lines and ensure the safety of both researchers and patients receiving products derived from these cultures.

In summary, combining cutting-edge diagnostics with strict quality control and ongoing research into EBV and OvHV-2 will be key to maintaining the reliability and safety of biotechnological innovations. By adopting these proactive strategies, we can minimize viral contamination, uphold research integrity, and improve the safety of therapeutic applications.
